# Commentary: The Brain Basis for Misophonia

**DOI:** 10.3389/fnbeh.2017.00111

**Published:** 2017-06-02

**Authors:** Arjan Schröder, Guido van Wingen, Nienke C. Vulink, Damiaan Denys

**Affiliations:** ^1^Department of Psychiatry, Academic Medical Center, University of AmsterdamAmsterdam, Netherlands; ^2^The Netherlands Institute for Neuroscience, An Institute of the Royal Netherlands Academy of Arts and SciencesAmsterdam, Netherlands

**Keywords:** misophonia, anger, disgust, fMRI, sound

In their recent study Kumar et al. (2017) investigated misophonia using functional and structural MRI. Their results show increased activity and connectivity between brain regions included in the “salience network,” notably the anterior insular cortex (AIC), which is involved in the processing of emotions. Heart rate and galvanic skin responses were increased during misophonic triggers, suggesting increased physical arousal. They postulate having found “*the brain basis for misophonia*” and that “*misophonia is attributed to particular sounds based on abnormal activation and functional connectivity of AIC*.” While we congratulate them on carrying out this novel study, we believe the above-quoted sentences are overstated because of important shortcomings.

Firstly, it is unclear whether participants in this study genuinely suffered from misophonia. Even though misophonia is not yet mentioned in the International Classification of Diseases (ICD) or Diagnostic and Statistical Manual of Mental Disorders (DSM), there is increasing evidence delineating it as a distinct psychiatric disorder with specific and well-defined diagnostic criteria (Edelstein et al., 2013; Johnson et al., 2013; Schröder et al., 2013; Schneider and Arch, 2015). Patients should be (1) obsessed with specific sounds, (2) experience intense anger, and consequently (3) avoid cue-related situations.

In the study of Kumar, subjects were selected based on one single unvalidated questionnaire, no structured psychiatric assessment was made, no involvement of psychiatrist or psychologist is presented, participants were not screened in a face-to-face interview, no information is provided about co-morbidity (subjects may have suffered from hyperacusis or borderline personality disorder, for example), neither use of psychopharmaceuticals or recreational drugs is mentioned. The authors did not make use of the aforementioned diagnostic criteria of misophonia. Hence, it is uncertain whether the brain differences may be attributed to misophonia.

Secondly, it is unclear whether the triggered emotions involve anger, which is essential for the diagnosis of misophonia (Schröder et al., 2013). In this study, participants were only asked to rate their annoyance, not their anger. The observed brain differences may therefore be correlated to general annoyance but not to specific anger. Again, in many other psychiatric disorders patients feel annoyed by sounds, e.g., in autism spectrum disorder and ADHD.

Thirdly, it is unclear because of the design of the study asking participants to visit the lab on two separate occasions, to what extent they have been sensitized to the sounds by repeated exposure. Sensitization implies that repeated exposure to a certain stimulus increases the response.

It cannot be ruled out that participants of the misophonic group were sensitized to the trigger sounds presented in this study.

In conclusion, though the study has triggered interesting discussions about the validity of misophonia, any statements about the “brain basis for misophonia” are, in our opinion, premature since there is lack of evidence that the participants really suffer from misophonia and that the triggered emotions are misophonia related.

## Author contributions

All authors listed, have made substantial, direct and intellectual contribution to the work, and approved it for publication.

### Conflict of interest statement

The authors declare that the research was conducted in the absence of any commercial or financial relationships that could be construed as a potential conflict of interest.
